# Electroconvulsive therapy modulates plasma pigment epithelium-derived factor in depression: a proteomics study

**DOI:** 10.1038/tp.2017.51

**Published:** 2017-03-28

**Authors:** K M Ryan, A Glaviano, S M O'Donovan, E Kolshus, R Dunne, A Kavanagh, A Jelovac, M Noone, G M Tucker, M J Dunn, D M McLoughlin

**Affiliations:** 1 Trinity College Institute of Neuroscience, Trinity College Dublin, Dublin, Ireland; 2 Department of Psychiatry, Trinity College Dublin, St. Patrick's University Hospital, Dublin, Ireland; 3 Conway Institute of Biomolecular and Biomedical Science, University College Dublin, Dublin, Ireland

## Abstract

Electroconvulsive therapy (ECT) is the most effective treatment for severe depression, yet its mechanism of action is not fully understood. Peripheral blood proteomic analyses may offer insights into the molecular mechanisms of ECT. Patients with a major depressive episode were recruited as part of the EFFECT-Dep trial (enhancing the effectiveness of electroconvulsive therapy in severe depression; ISRCTN23577151) along with healthy controls. As a discovery-phase study, patient plasma pre-/post-ECT (*n*=30) was analyzed using 2-dimensional difference in gel electrophoresis and mass spectrometry. Identified proteins were selected for confirmation studies using immunodetection methods. Samples from a separate group of patients (pre-/post-ECT; *n*=57) and matched healthy controls (*n=*43) were then used to validate confirmed changes. Target protein mRNA levels were also assessed in rat brain and blood following electroconvulsive stimulation (ECS), the animal model of ECT. We found that ECT significantly altered 121 protein spots with 36 proteins identified by mass spectrometry. Confirmation studies identified a post-ECT increase (*P*<0.01) in the antiangiogenic and neuroprotective mediator pigment epithelium-derived factor (PEDF). Validation work showed an increase (*P*<0.001) in plasma PEDF in depressed patients compared with the controls that was further increased post-ECT (*P*=0.03). PEDF levels were not associated with mood scores. Chronic, but not acute, ECS increased PEDF mRNA in rat hippocampus (*P*=0.02) and dentate gyrus (*P*=0.03). This study identified alterations in blood levels of PEDF in depressed patients and further alterations following ECT, as well as in an animal model of ECT. These findings implicate PEDF in the biological response to ECT for depression.

## Introduction

Electroconvulsive therapy (ECT) is the most effective treatment for severe, often treatment-resistant, and sometimes life-threatening, depression.^1, 2^ Of note, despite being in use since 1938, its mechanism of action is still not fully understood. Electroconvulsive stimulation (ECS), the animal model equivalent of ECT, has helped us begin to elucidate the neurobiological mechanisms of ECT. In this regard, ECS has been shown to bring about a wide range of molecular and cellular changes in the brain that may be involved in its action. These include changes in monoamine neurotransmitters, synaptic plasticity, neurogenesis, hypothalamic–pituitary–adrenal-axis activity and the neuroinflammatory response.^3, 4, 5, 6, 7^ However, the clinical utility of measuring molecular changes in the brains of patients is limited by the invasive nature of obtaining biopsies or cerebrospinal fluid (CSF). Importantly, blood has previously been shown to reflect brain-related protein levels, and it is known that exchanges can occur between the central nervous system and the periphery in both healthy and disease states.^8, 9^ Thus, peripheral blood represents a more clinically accessible tissue and provides a more minimally invasive, acceptable and inexpensive option for routine testing.

One unbiased screening approach to identify novel candidate protein changes in patient blood is to use proteomic methods, that is, studying all proteins expressed in a cell, tissue, or organism at a given moment under determined conditions.^10^ Using proteomic analyses, we and others have shown that ECS induces a wide range of changes in rodent brain and blood proteomes, including changes in acute phase proteins, carrier proteins, cell signaling proteins, neurotrophic factors, and persisting changes in cytoskeletal and metabolism-related proteins.^11, 12, 13, 14^ However, to our knowledge, there has only been one relatively small pilot study (*n*=12) carried out to date investigating the serum proteome of depressed patients receiving treatment with ECT.^15^ After a single ECT treatment, changes in serum proteins and small molecules were identified, with changes in platelet factor 4 significantly related to symptom improvement. Interestingly, after a full course of ECT changes in serum protein levels were also found, but these had no relation to symptom improvement.^15^

We have now conducted a larger proteomic discovery-phase study using 2-dimensional difference in gel electrophoresis (2D-DIGE) and mass spectrometry. Plasma samples taken from severely depressed patients before (pre-) and following (post-) a course of ECT and who attained remission following treatment were analyzed to identify plasma protein changes associated with ECT for depression. These findings were then confirmed using immunodetection methods. Validation investigations were also carried out in samples from a second cohort of severely depressed patients pre- and post-ECT alongside age- and sex-matched healthy controls. We additionally extended our analyses to an animal model of ECT. Understanding such protein changes may provide new insights into the molecular response to ECT and its mechanism of action.

## Materials and methods

### Human participants

The study was approved by the St Patrick's University Hospital Research Ethics Committee. All participants provided written informed consent. All depressed patients were recruited as part of the EFFECT-Dep Trial (Enhancing the Effectiveness of ECT in Severe Depression; http://www.isrctn.com/ISRCTN23577151).^16^ The EFFECT-Dep Trial was a pragmatic, patient- and rater-blinded, non-inferiority trial of patients with major depression that compared the effects of twice-weekly moderate dose bitemporal (1.5 × seizure threshold) and high-dose unilateral (6 × seizure threshold) ECT in real-world practice. Recruitment was conducted between 2008 and 2012 in St Patrick's Mental Health Services, an independent non-profit organization that runs Ireland's largest ECT clinic (http://www.stpatricks.ie/). For the main study, 138 participants who met diagnostic criteria for a major depressive episode were recruited and randomly allocated to a treatment group prior to the first ECT session. To be included in the trial, participants had to be over 18 years old, be referred for ECT for treatment of a major depressive episode as diagnosed by the Structured Clinical Interview for DSM-IV Axis I Disorders^17^ and have a pre-treatment 24-item Hamilton Depression Rating Scale (HAM-D) score ⩾21.^18^ Patients were not eligible to take part in the trial if they had the following: conditions rendering them unfit to receive general anesthesia or ECT; ECT in the previous 6 months; a history of schizophrenia, schizoaffective disorder, or a neurodegenerative or other neurological disorder; alcohol or substance abuse in the previous six months; an involuntary status; an inability or refusal to provide consent.

ECT was administered twice weekly with hand-held electrodes as previously described, using methohexital (0.75–1 mg kg^−1^) anesthesia and succinylcholine (0.5–1 mg kg^−1^) as a muscle relaxant.^16, 19^ Patients were maintained on their usual medication during the course of ECT. Remission criteria were a 60% decrease in baseline HAM-D score and an end-of-treatment HAM-D score ⩽10 for two consecutive weeks. Fasting blood samples were taken at 0730–0930 hours before the first ECT treatment and 1–3 days after the final treatment. Overall, 10 ml of peripheral blood were collected into K_2_EDTA tubes (BD, Oxford, UK). Tubes were centrifuged at 2000 r.p.m. for 10 min following manufacturer's guidelines, and plasma was collected and stored in aliquots at −80 °C until analysis.

Healthy controls were recruited through advertisement in local newspapers and social media. Fasting control blood samples were taken between 0730 and 0930 hours on assessment days.

### Study design

For the discovery-phase proteomics study, we selected ECT remitters (*n*=30; Table 1) recruited in the first half of the trial from 2008 to 2010 to enrich the sample for the therapeutic effect of ECT. Pre- and post-ECT plasma samples were compared to identify proteome changes and the same samples were used for confirmation studies.

Plasma samples from a separate group of depressed patients participating in the EFFECT-Dep Trial and treated with ECT (*n*=57), as well as from age- and sex-matched healthy controls (*n*=43; see Supplementary Table 1 for demographic and clinical characteristics) were used to validate confirmed proteins.

Laboratory analyses were performed with the investigator unblinded to the group the samples belonged to (that is, control, depressed pre-ECT, depressed post-ECT) but blinded to all clinical and demographic data.

### 2D-DIGE and mass spectrometry

Plasma samples were separated by 2D-DIGE as previously described.^11, 12^ Prior to 2D-DIGE analysis, 85–90% of the six most abundant proteins found in human plasma (albumin, IgG, IgA, transferrin, haptoglobin and antitrypsin) were removed, yielding two protein fractions from each plasma sample: low-abundance and high-abundance proteins. Differentially expressed protein spots were targeted for identification by mass spectrometry based on their statistical significance (*P*<0.05), their location/shape following a comparison of CyDye-labeled gel images, and manual inspection of silver-stained preparative gels. Only spots that could easily be excised were considered for mass spectrometry as it is difficult to excise smaller spots and ensure that the excised area does not incorporate proteins from adjacent spots. For a detailed description, see the Supplementary Materials and Methods.

### Gene ontology analysis

Uniprot accession numbers of all identified proteins were uploaded into the Database for Annotation, Visualization and Integrated Discovery (DAVID) (http://david.abcc.ncifcrf.gov)^20, 21^ as described previously.^12^ See Supplementary Materials and Methods for further details.

### Confirmation and validation of differentially expressed proteins

Proteins were selected for confirmation plasma studies from spots found to be altered by a fold-change ⩾1.1, based upon a 2-peptide minimum criterion and, in the first instance, having been previously implicated in depression^22, 23, 24^ or antidepressant treatment.^11, 25^ Three proteins, for which immunoassay kits were readily available, were selected for confirmation and validation studies. PEDF and haptoglobin were selected from the proteins identified in the low-abundance gels, whereas serotransferrin was selected from the proteins identified in the high-abundance gels. The Phase Range Haptoglobin kit (TP801; TriDelta Development, Kildare, Ireland) was used to determine haptoglobin concentrations. Sandwich ELISA kits were used to measure serotransferrin (CSB-E13761h; Cusabio Biotech, Wuhan, China) and pigment epithelium-derived factor (PEDF) (CYT420; Millipore, Cork, Ireland) concentrations. The intra-assay % coefficient of variance for all assays was <10, whereas the inter-assay % coefficient of variance for PEDF was <20.

### Analysis of PEDF mRNA levels

To assess the effects of ECS on PEDF mRNA levels in brain, rats (*n*=7–8 per group) were treated acutely (1 × ) or chronically (10 × ) with ECS, and brain and blood samples were harvested as previously described^26^ (Supplementary Materials and Methods). mRNA extractions, cDNA synthesis and qRT-PCR were carried out as described in the Supplementary Materials and Methods.

### Statistical analysis

One-way ANOVA was applied by Progenesis SameSpot software (Nonlinear Dynamics, Newcastle upon Tyne, UK) to determine differences in normalized protein spot expression pre-/post-ECT. A value of *P*<0.05 was considered statistically significant.

Confirmation and validation study data analyses were performed using SPSS version 22.0 (IBM, New York, NY, USA). Categorical data were tested using χ^2^ tests unless otherwise specified. All data were tested for normality using a Shapiro–Wilk test and log-transformed where necessary. Data with normal distributions were analyzed using a two-sided Student's *t*-test or analysis of variance (ANOVA). Non-parametric data were analyzed with the Wilcoxon-signed rank test for paired comparisons. Correlation analyses were performed using Pearson's correlation coefficient *r* or Spearman's rank correlation coefficient *ρ*. With regard to potential confounders, patients and controls were matched by age and sex. Other potential confounders, such as body mass index (BMI), smoking, level of alcohol consumption and socio-economic group were considered to be confounders if they differed between groups (depressed and controls) and correlated with the dependent variable (PEDF values).

Changes over time were analyzed using a mixed-design ANOVA with time as the within-subjects factor and remission status, polarity, presence of psychosis and electrode position as between-subjects factors. Data are presented as means with standard error of the mean (s.e.m.). A *P*-value of <0.05 was considered statistically significant.

## Results

### 2D-DIGE and mass spectrometry

A total of 47 protein spots were significantly altered following ECT in the low-abundance protein gels (Supplementary Figure 1), from which 25 spots were selected for identification using mass spectrometry and 32 proteins were ultimately identified. In high-abundance gels, 74 protein spots were significantly altered following ECT (Supplementary Figure 2), from which 20 spots were selected for identification by mass spectrometry and four proteins were ultimately identified. Several of these proteins were identified in multiple spots in both sets of gels. The proteins identified are listed in Supplementary Tables 2 and 3.

### Gene ontology analysis

DAVID analysis categorised low-abundance proteins according to the gene ontology terms biological process, molecular function and cellular component. The number of proteins annotated to each enrichment term is expressed as a percentage of the total number of proteins identified in each category (Supplementary Figure 3). The *P*-value, false discovery rate and accession numbers of the proteins classified by DAVID are listed for low- and high-abundance proteins in Supplementary Tables 4 and 5. A single KEGG pathway, complement and coagulation cascades (*P*=1.37E−9; false-discovery rate *P*=1.14E−6), was identified from the low-abundance proteins, whereas no significant KEGG pathway was identified from high-abundance proteins.

### Confirmation of protein changes

**Figure 1:**
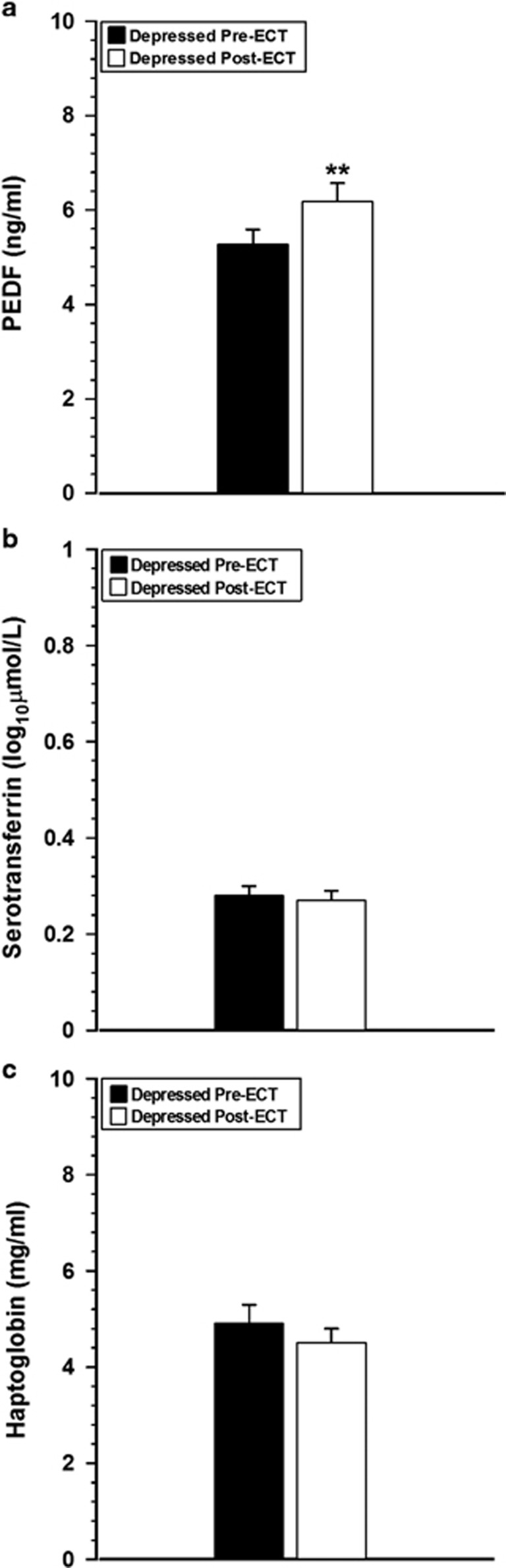
Confirmation studies of differentially expressed proteins. Plasma samples collected from depressed patients (*n*=30) before and after completing a course of ECT and included in the discovery-phase proteomic study were used for confirmation studies using ELISA and colorimetric assays. (**a**) Plasma PEDF concentrations were significantly increased following ECT. There was no change in the concentration of (**b**) serotransferrin or (**c**) haptoglobin following ECT. Data are expressed as means±s.e.m. **P<0.01 versus pre-ECT counterpart (PEDF, serotransferrin: paired *t*-test; haptoglobin: Wilcoxon-signed rank test). ECT, electroconvulsive therapy; PEDF, pigment epithelium-derived factor.

The proteins selected for confirmation studies were PEDF, serotransferrin and haptoglobin. PEDF data were normally distributed, whereas data for serotransferrin and haptoglobin were not. Serotransferrin data attained normality following log transformation and so parametric testing was used to analyze both serotransferrin and PEDF. Haptoglobin failed to attain normality, even after log transformation; therefore, non-parametric testing was used. PEDF concentrations increased (Figure 1a; *P*=0.003) following ECT. There was no change in serotransferrin (Figure 1b; *P*=0.36) or haptoglobin (Figure 1c; *P*=0.49) levels.

### Validation of changes in plasma PEDF protein levels

**Figure 2:**
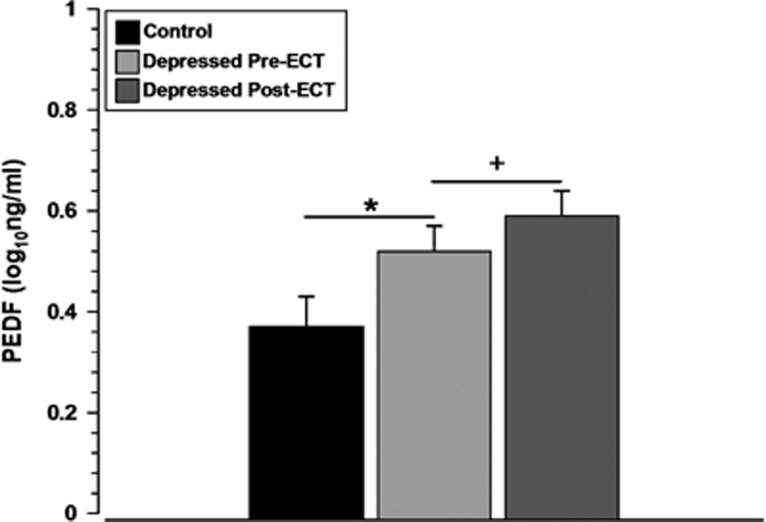
Plasma PEDF levels during a major depressive episode and following ECT. Validation studies show plasma PEDF is greater in depressed patients (*n*=57) compared with the healthy control participants (*n*=43) and is increased following ECT. Data are expressed as mean log_10_ ng ml^−1^±s.e.m. *P<0.05 versus healthy controls (unpaired *t*-test), ^+^*P*<0.05 pre-ECT versus post-ECT-depressed patients (paired *t*-test). ECT, electroconvulsive therapy; PEDF, pigment epithelium-derived factor.

As we confirmed an increase in levels of PEDF following ECT, we sought to examine plasma PEDF levels in depressed patients pre-ECT (*n*=57) compared to age- and sex-matched healthy controls (*n*=43) and validate the changes in PEDF observed following ECT in a larger independent sample (Figure 2). PEDF levels were significantly greater in depressed patients compared with the controls (Figure 2a; *P*<0.05). There was no significant association between PEDF levels and factors having a potential effect on baseline PEDF levels such as BMI^27^ (*r*=0.15, *P*=0.15), alcohol^28^ (*ρ*=−0.13, *P*=0.19) or smoking^29^ (*P*=0.21).

In depressed patients, PEDF levels were significantly increased following ECT (*P*=0.03). A mixed-design ANOVA indicated no significant between-subjects differences based on remission status (F_(1,55)_=0.48, *P*=0.49), depression polarity (F_(1,55)_=0.32, *P*=0.57), presence of psychosis (F_(1,55)_=0.01, *P*=0.94) or electrode placement (F_(1,55)_=0.15, *P*=0.70).

### PEDF and clinical outcome

Our discovery-group consisted only of remitters, whereas our validation group contained 16% remitters. Therefore, to explore the relationship between plasma PEDF levels and clinical outcome, we pooled the confirmation and validation PEDF protein data (*n*=87; Supplementary Table 6). This pooled group had a mean age of 55.5±14.6 years, mean baseline HAM-D of 30.7±7.1, mean post-ECT HAM-D of 11.2±8.5, and was comprised of 46% remitters, which is similar to the total EFFECT-Dep Trial group.^16^ Baseline levels of PEDF did not correlate with pre-ECT HAM-D scores (*r*=0.11, *P*=0.32) nor did changes in PEDF levels correlate with changes in HAM-D (*r*=0.05, *P*=0.67). A mixed-design ANOVA showed no effect of remission status on the change in PEDF levels (F_(1,85)_=1.23, *P*=0.23).

### PEDF mRNA changes in ECS-treated rats

**Figure 3:**
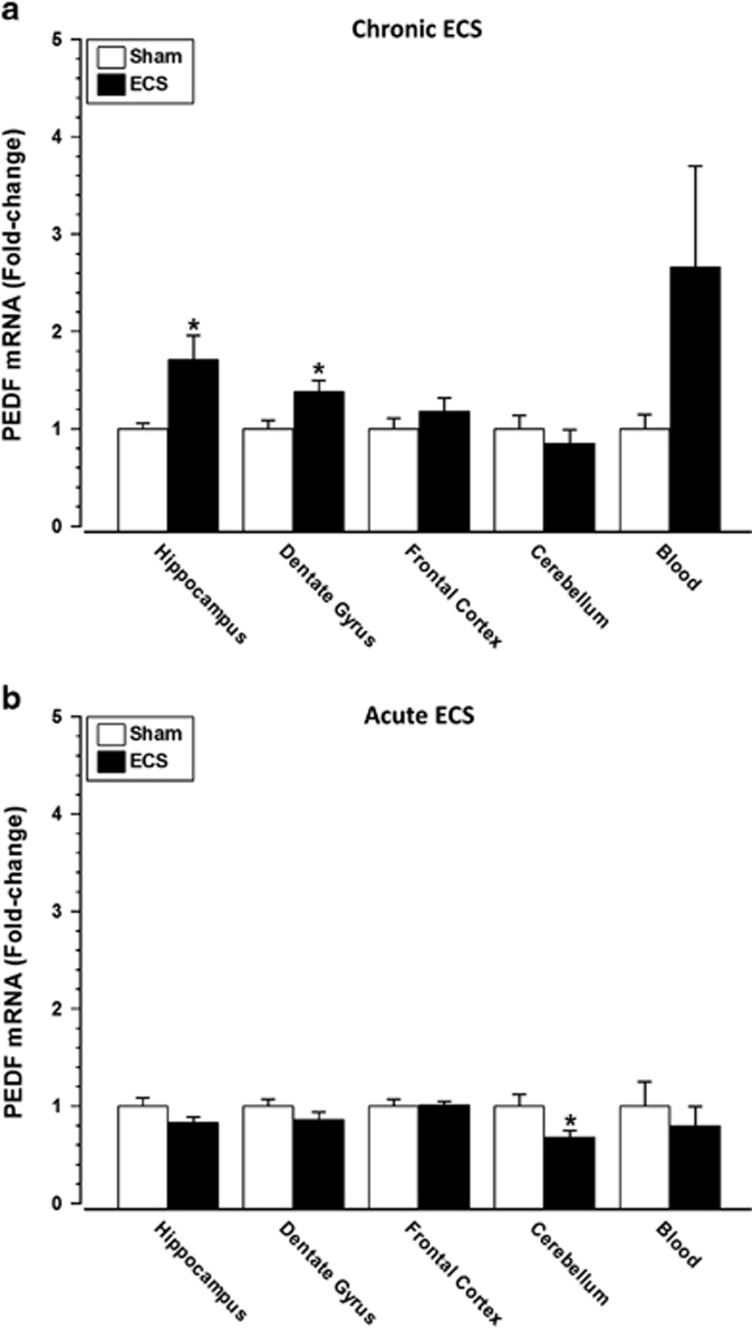
PEDF mRNA changes in rat brain and blood following acute and chronic ECS. Rats were treated chronically (10 × ) or acutely (1 × ) with ECS and killed 4 h post treatment. (**a**) PEDF mRNA levels were significantly increased following chronic (10 × ) ECS in the hippocampus (*n*=7 per group) and dentate gyrus (*n*=7–8 per group) but not in the frontal cortex (*n*=7 per group), cerebellum (*n*=7–8 per group) or blood (*n*=5–6 per group). (**b**) PEDF mRNA levels were significantly decreased following acute (1 × ) ECS in the cerebellum (*n*=8 per group) but not in the hippocampus (*n*=8 per group), dentate gyrus (*n*=8 per group), frontal cortex (*n*=8 per group) or blood (*n*=6–7 per group). Data expressed as mean±s.e.m. *P<0.05 versus Sham ECS control (Student's *t*-test). ECS, electroconvulsive stimulation; PEDF, pigment epithelium-derived factor.

The source of PEDF in blood is not clear. We therefore used ECS in naive rats to assess the effects of acute (1 × ) and chronic (10 × ) ECS sessions on brain PEDF mRNA. There was no difference in body weight between real and sham-treated ECS groups throughout the chronic treatment period (F_(9,117)_=1.66, *P=*0.11). PEDF mRNA levels significantly increased in the hippocampus (*P*=0.02) and dentate gyrus (*P*=0.03) of rats treated chronically with ECS, with no change observed in the frontal cortex (*P*=0.26) or cerebellum (*P*=0.48) (Figure 3a). Although PEDF mRNA levels increased in blood following chronic ECS, the change was not statistically significant (Figure 3a: *P*=0.18). Following acute ECS, PEDF mRNA levels were significantly decreased in the cerebellum (*P*=0.04), but no change was observed in the hippocampus (*P*=0.16), dentate gyrus (*P*=0.21), frontal cortex (*P*=0.92) or blood (*P*=0.55) (Figure 3b).

## Discussion

The results of our proteomic screen showed significant changes in high- and low-abundance plasma proteins in severely depressed patients who remitted following ECT, with four proteins identified by mass spectrometry in high-abundance gel spots and 32 proteins in low-abundance gel spots. Alterations in these proteins particularly implicate the immune response. As multiple proteins were identified in the majority of spots using 2D-DIGE, confirmation of proteomic results using a more targeted immunodetection approach was necessary.^30^

For initial confirmation studies, we selected three proteins that have previously been implicated in depression^22, 23, 24^ or antidepressant treatments,^11, 25^ which adds to the biological plausibility that changes in these proteins might be relevant. On this basis, we selected serotransferrin, haptoglobin and PEDF; ongoing work is being carried out to investigate changes in other proteins detected. Serotransferrin is a negative acute phase protein that is decreased in first-onset, drug naive major depressive disorder (MDD) patients.^23^ Haptoglobin is a positive acute phase protein that is increased in MDD^24^ and decreased in rat plasma following chronic ECS.^11^ PEDF is a serine protease inhibitor that exhibits antiangiogenic, neurotrophic and neuroprotective effects.^31^ Previous studies found PEDF to be increased in the CSF of MDD patients, although it is not clear whether these patients were taking antidepressants,^22^ and upregulated in mouse hippocampus following chronic fluoxetine exposure.^25^ We found no change in serotransferrin or haptoglobin but PEDF protein was significantly increased following ECT.

Results from our discovery and confirmation studies indicate that PEDF is a novel contributor in the biological response to ECT. It is imperative to ascertain whether changes identified in a small sample set are also found in a different sample.^32^ We therefore validated these findings in a larger cohort of patients and found that plasma PEDF protein levels were significantly increased in depressed patients compared with the healthy controls, with this increase augmented by ECT. Previous reports indicate that BMI,^27^ alcohol^28^ and smoking^29^ may affect baseline levels of PEDF. However, we detected no significant effect of these factors. All of our patients were receiving pharmacological treatment as usual during the course of ECT. Thus, it is possible that the increased baseline levels of PEDF observed in patients is an effect of the pharmacological antidepressant treatments they were receiving, and that ECT acts to enhance this antidepressant effect. Thus, further studies involving samples from drug naive patients with depression before and following antidepressant treatments are required to fully evaluate PEDF levels in patients. In addition, our patients were also receiving anesthesia and muscle relaxants during ECT, which might also affect PEDF levels. Thus, in an effort to clarify the effects of ECT on PEDF, we examined PEDF mRNA expression in rats treated with ECS. Although patients received other antidepressant treatments, anesthesia and muscle relaxants during the course of ECT, our ECS-exposed rats did not. PEDF mRNA was found to be increased following chronic, but not acute, ECS, an effect that was selective to the hippocampal formation with a related possible increase in blood. Therefore, ECS/ECT itself does indeed induce alterations in PEDF levels.

Although PEDF was altered in depression and by ECT, we found no association with mood scores. A previous proteomic report from Stelzhammer *et al.*^15^ showed similar findings following a course of ECT. After six ECT sessions, patients showed significant improvements in scores on the HAM-D scale. In addition, significant changes were seen in four serum proteins after chronic ECT, that is, alpha1-antichymotrypsin, apolipoprotein A2, clusterin and, interestingly, serotransferrin. However, comparable to our results with PEDF, changes in these proteins were not related to symptom improvement. These results emulate recent findings with the neurotrophin brain-derived neurotrophic factor (BDNF). Emerging data led to the theory that ECT may induce its clinical effect by increasing the expression of BDNF, which is decreased in depression, and thereby boosting neuronal plasticity.^33, 34^ A recent meta-analysis showed that in pre-clinical studies ECS-induced BDNF in the brain is indeed associated with behavioral changes in rodents, albeit slightly so.^33^ In contrast, however, a meta-analysis of the human data available shows that BDNF is increased in blood, in particular plasma, following treatment with ECT but this is not associated with the clinical efficacy of ECT.^33^ This suggests that changes in proteins such as PEDF may be important in the pathogenesis of depression and in the molecular mechanisms of action of ECT, but also that they do not necessarily correspond with the therapeutic response to ECT. Interestingly, PEDF levels were not normalized following ECT treatment but instead were increased further, suggesting that PEDF may act as an intermediary molecule in the mechanism of ECT. Ultimately, this points to a complex relationship between PEDF and mood, which requires further investigation and clarification.

PEDF is an endogenously expressed 418 amino acid 50 kDa glycoprotein encoded by the serpin peptidase inhibitor, clade F, member 1 (*SERPINF1*) gene on chromosome 17p13. It was first identified as a protein secreted by human fetal eye pigment epithelium.^35^ PEDF is widely expressed and has been detected in blood,^36^ CSF,^37^ aqueous humor^38^ and other body fluids at physiologically relevant concentrations.^31^ It belongs to the serine protease inhibitor (serpin) family but, although it has structural homology with other serpins, it is non-inhibitory, possibly because of differences in its reactive center loop, which determines functional specificity.^39^ PEDF primarily signals through the PEDF receptor (PEDF-R), a transmembrane protein encoded by Patatin-like phospholipase domain containing 2 (*PNPLA2*) present on the cell surface and which exhibits phospholipase A2 (PLA2) activity.^40^ PNPLA2 is expressed on blood cells,^41, 42^ adipocytes,^43^ and also on neurons^44^ and glia^45^ in the brain. Peptides derived from PEDF have neuroprotective or antiangiogenic functions.^39^ PEDF binding to PEDF-R stimulates PLA2 activity causing release of fatty acids, either omega-3 docosahexanoic acid or omega-6 arachidonic acid, and lysophosphatidic acid from membranous phospholipids. Elevated docosahexanoic acid can antagonize arachidonic acid, which is tumorigenic in nature, and increases the release of the fatty acid derivative neuroprotectin D1, which may mediate PEDF's neurotrophic and anti-inflammatory activities.^31^ PEDF is also known to be antiangiogenic and antitumorigenic, and to function in tissue homeostasis.^46^ This appears to occur through a counterbalance with vascular endothelial growth factor, which is pro-angiogenic^47^ and has previously been implicated in depression.^48^ Notably, PEDF is downregulated in many cancers.^31^

ECS/ECT alters the expression of numerous neurotrophins^26, 49, 50^ and PEDF is another neurotrophin upregulated following ECS/ECT, which may add further evidence to the neurotrophic hypothesis of depression.^51^ The neurotrophic and neuroprotective activity of PEDF is suggested to come from a 44-amino-acid fragment corresponding to positions 78–121.^52^ PEDF also promotes activation of the immune-related transcription factor NFkB,^39^ which in turn can induce an increase in antiapoptotic proteins, pro-survival genes or neurotrophins in neuronal cells.^39^ PEDF promotes neurite outgrowth in Y79 retinoblastoma cells^53^ and developing spinal motor neurons,^54^ and has also been shown to prevent neuronal atrophy in various models.^39^ Interestingly, recombinant human PEDF induces pro-inflammatory cytokine and chemokine expression in microglia^55, 56^ and astrocytes^57^ *in vitro*, suggesting that PEDF may act as a neuroimmune modulator in the CNS. Taken together, PEDF may mediate its effects through neurotrophic, antiangiogenic and/or immunological mechanisms, all of which are relevant to depression neurobiology.^58^ As these systems influence each other,^4, 59, 60^ it is likely that a complex interplay between them is responsible for the effects of PEDF in depression and the antidepressant response; however, further work is requilred to determine the precise mechanisms involved.

The source of PEDF in the plasma is unknown. Previous studies have indicated that PEDF is found in astroglial exosomes,^61^ nanometer-sized vesicles ~30–100 nm in diameter that are derived from endocytic compartments and are secreted from the cell. Exosomes are found to contain the proteins, lipids and RNA of their parent cells. In the brain, exosome release is associated with depolarization and neurotransmitter signaling. Notably, evidence suggests that exosomes from neurons and glia can be detected in the CSF and can also pass from the brain into the circulation via the blood–brain barrier.^9^ However, the mechanism for this is not fully understood. Such studies suggest that the plasma PEDF in our study may be coming from the brain although further work is required to fully investigate this possibility.

The precise mechanisms responsible for the changes in PEDF seen in the present study remain to be determined. We speculate that the increase in PEDF is occurring due to an alteration in transcription, a decrease in turnover of the protein through either an increase in its half-life, decreased degradation or post-translational modifications, such as glycosylation^36^ or phosphorylation.^62^

A limitation of this study is that, as already mentioned, our patients were all receiving pharmacological treatment as usual during ECT. Thus, it cannot be ruled out that the increase in PEDF observed in depressed patients is an effect of antidepressant treatment and that ECT is acting to enhance this antidepressant effect. A previous study reporting increased basal levels of PEDF in CSF of depressed patients did not detail whether patients were receiving antidepressants or not.^22^ Moreover, our patients were receiving anesthesia and muscle relaxants during ECT. In an effort to clarify the effects of ECT on PEDF, we examined PEDF mRNA expression in rats treated with ECS. As already mentioned, although patients received other antidepressant treatments, anesthesia and muscle relaxants during the course of ECT, our ECS-exposed rats did not. We found an increase in PEDF mRNA following chronic, but not acute, ECS that was selective to the hippocampal formation with a related possible increase in blood. However, a limitation of this work is that we did not analyse PEDF protein levels in the ECS model as tissue samples were unavailable. Importantly, there was no difference in the body weight of rats treated with real or sham ECS indicating that this treatment regimen was not stressful to the animals. This suggests that stress itself did not elevate PEDF. These findings provide further evidence that chronic ECT induces changes in human peripheral PEDF, and that similar changes also occur in the brain of an ECT rat model. Future studies will need to include analyses of PEDF protein levels and also models of depression. Moreover, further work is required to examine levels of PEDF in unmedicated depressed patients, both at baseline compared with the controls and pre-/post-antidepressant treatment, to gain better insight into the changes that occur in PEDF in depression and the therapeutic response. A further limitation of this study is the time point at which plasma samples were collected post-ECT, which ranged from 1–3 days. This may have led to variation in our dataset resulting in the lack of correlation of PEDF changes with mood score changes. Bumb *et al.*^63^ have previously reported that peak BDNF levels only occur after a latency of a number of days following a course of ECT. Moreover, an animal study of ECS showed that there is a time delay in the increase in peripheral BDNF levels following the ECS-induced increase in central levels, indicating that it takes time for peripheral levels to equilibrate with central levels.^64^ Thus, to fully evaluate the impact of the time point for blood collection on PEDF levels a time-course study will be useful in the future; however, this was beyond the scope of the current work.

Our results provide some novel insights into the biological response to ECT for depression and possibly its mechanism of action. Evidence now exists to suggest that alterations in PEDF are a common molecular response to antidepressant treatments. Further work is required to fully explore the role of PEDF in both depression and antidepressant treatments.

## Figures and Tables

**Table 1:** Demographic and clinical characteristics of the ECT remitter participants in the gel-based proteomic discovery-phase study

*Characteristic*	*ECT remitters (*n*=30)*
Age, mean (s.d.), years	60 (13.5)
	
*Sex, no. (%)*	
Male	12 (40)
Female	18 (60)
	
BMI, mean (s.d.)	25.5 (4.4)
Alcohol median units per week (range)	1 (0–40)
Smokers, no. (%)	11 (36.7)
	
*Socio-economic group, no. (%)*	
1	7 (23.3)
2	8 (26.7)
3	7 (23.3)
4	2 (6.7)
5	6 (20)
	
Bipolar depression, no. (%)	5 (16.7)
Psychotic depression, no. (%)	7 (23.3)
	
*Medications, no. (%) taking*	
SSRI	6 (20)
SNRI	20 (66.7)
TCA	7 (23.3)
Mirtazapine	11 (36.7)
Trazodone	3 (10)
Lithium	13 (43.3)
Sodium valproate	3 (10)
Antipsychotics	19 (63.3)
Benzodiazepines	23 (76.7)
Non-benzodiazepine hypnotics	16 (53.3)
	
Baseline HAM-D, mean (s.d.)	31.8 (6.9)
Post-ECT HAM-D, mean (s.d.)	4.9 (3.6)
	
*Electrode placement, no. (%)*	
Unilateral	15 (50)
Bitemporal	15 (50)
	
Number of ECT sessions, mean (s.d.)	7 (2.30)
Remitters, no. (%)	30 (100)

Abbreviations: BMI, body mass index; ECT, electroconvulsive therapy; HAM-D, Hamilton Depression Rating Scale, 24-item version; SNRI, serotonin-norepinephrine reuptake inhibitor; SSRI, selective serotonin reuptake inhibitor; TCA, tricyclic antidepressant.
