# Supplementary material for: Electroconvulsive therapy modulates plasma pigment epithelium-derived factor in depression: a proteomics study
Source: Transl Psychiatry. 2017 Mar 28;7(3):e1073–. doi: 10.1038/tp.2017.51 (PMC5404616; doi:10.1038/tp.2017.51)
Supplement: Supplementary Information [file tp201751x1.docx]

**Electroconvulsive therapy modulates plasma pigment epithelium-derived factor in depression: a proteomics study**

**Supplementary material and methods**

**Sample preparation**

Plasma samples were separated by 2-dimensional difference gel electrophoresis (2D-DIGE) as previously described.^1, 2^ Prior to 2D-DIGE analysis, approximately 85–90% of the six most abundant proteins found in human plasma (albumin, IgG, IgA, transferrin, haptoglobin, and antitrypsin) were removed using the Multiple Affinity Removal System (MARS; Agilent Technologies, USA) in the Vision WorkStation BioCAD® Family High Performance Liquid Chromatography (HPLC; Applied Biosystems, USA). This resulted in two protein fractions from each plasma sample, one containing low-abundance proteins and the other high-abundance proteins.

Following immunodepletion, samples were concentrated and ‘cleaned-up’ through acetone precipitation, with four volumes of cold acetone added to one volume of each sample. Samples were incubated at -70ºC for 10 minutes and at -20ºC for a further 90 minutes and centrifuged at 15,000 x g for 15 minutes at 4ºC. Further impurities were eliminated to avoid first dimension streaking using a commercial kit (2D Clean-Up Kit, GE Healthcare, UK). The resulting pellet was air-dried and then resuspended in 50 µl of DIGE Lysis Buffer (pH 8.0–9.0) [9.5 M Urea (USB Corporation, USA), 2% CHAPS (USB Corporation, USA), 20 mM Tris pH 8.0 (USB Corporation, USA)]. Protein concentration was determined by Bradford protein assay.

Samples were normalized to a concentration of 5 µg/µl. The pH of all samples was measured using pH test strips (Sigma, USA) and fell within the recommended range of pH 8.0–9.0.

Samples were CyDye-labelled according to manufacturer’s instructions (CyDye kit, GE Healthcare, UK). Briefly, 50 µg of protein were labelled with 400 pmol of cyanine (Cy) dye prepared in dimethylformamide. Individual pre- and post-ECT samples were labelled with Cy5. Cy3 was used to label the internal standard, a pool consisting of an equal volume of all samples. The labelled samples were left on ice for 30 minutes in the dark before 1 µl of 10 mM Lysine (Sigma, UK) was added to stop the reaction.

One volume of 2X Dilution Buffer [9.5 M Urea (USB Corporation, USA), 2% CHAPS (USB Corporation, USA), 2% Dithiothreitol (DTT) (USB Corporation, USA), 1.6% Pharmalyte (Pharmacia, UK)] was added to each sample. Each Cy5 labelled sample was pooled with an equal volume of Cy3 labelled internal standard and made up to a final volume of 450 µl with rehydration buffer [8 M Urea (USB Corporation, USA), 0.5% CHAPS (USB Corporation, USA), 0.2% DTT (USB Corporation, USA), 0.2% Pharmalyte (Pharmacia, UK), Bromophenol Blue (Sigma, UK)].

**2D-DIGE**

For first dimension separation, samples were rehydrated overnight on immobilized pH gradient (IPG), 24 cm Immobiline Dry-Strips (pH 4–7) (GE Healthcare, UK). Isoelectric focusing was performed using Ettan IPGphor 3 (GE Healthcare, UK) according to the following protocol: 3500 V for 75000 Vh; 8000 V for 10 minutes; 8000 V for 1 h.

IPG strips were equilibrated in equilibration buffer [6 M Urea (USB Corporation, USA), 50 mM Tris HCl pH 8.8, 30% (v/v) Glycerol (USB Corporation, USA), 2% (w/v) Sodium Dodecyl Sulfate (SDS) (USB Corporation, USA)] containing 10 mg/ml dithiothreitol (USB Corporation, USA) for 15 minutes followed by immersion in equilibration buffer containing 25 mg/ml iodoacetamide (USB Corporation, USA) for 15 minutes.

Proteins were separated in the second dimension overnight on 12% SDS-PAGE gels in SDS electrophoresis buffer [(25 mM Tris (USB Corporation, USA), 192 mM Glycine (USB Corporation, USA, 0.1% (w/v) SDS (USB Corporation, USA), Bromophenol Blue (Sigma, UK)] at 0.2 W per gel until the dye band ran off the gel front.

**Gel image analysis**

Gels were scanned using the Typhoon 9410 scanner (Amersham Biosciences). The final scan was conducted at 100 µm (pixel). Approximately 1700 spots were observed in high abundance gels and 950 in low abundance gels. The gels were subjected to quantitative computer analysis to detect differentially expressed spots with Progenesis SameSpots software (Nonlinear Dynamics, UK). Analysis was carried out on the log_10_ transformed standardized spot abundance. The normalized spot volumes were analyzed using one-way ANOVA to compare treatment and control groups. Changes in spot abundance were considered statistically significant at *P*<0.05.

**Spot excision and tryptic digest**

Protein spots were targeted for identification by mass spectrometry based on their statistical significance (*P*<0.05) and their location following a comparison of CyDye-labelled gel images and manual inspection of silver-stained preparative gels. Only spots which could easily be excised were considered for mass spectrometry as it is difficult to excise smaller spots and ensure that the excised area does not incorporate proteins from adjacent spots. Preparative gels (1.5 mm) were run with 400 µg of protein from the pooled internal standard sample and the gels were stained with a mass spectrometry compatible silver stain (GE Healthcare, UK). Spots were excised and destained in an equal volume of 30 mM potassium ferricyanide (Sigma, UK) and 100 mM sodium thiosulphate (Sigma, UK). Gel spots were dehydrated and trypsin digested in lyophilized trypsin (20 ng/µl) (Promega, USA) overnight at 37°C. Tryptic peptides were extracted three times with a 70% acetonitrile (Fisher Scientific, UK) and 5% formic acid (Sigma, UK) solution. Combined extracts were then dried and stored at -20°C until required for mass spectrometry.

**Mass spectrometry**

The samples were run on a Thermo Scientific LTQ ORBITRAP XL mass spectrometer connected to a Dionex Ultimate 3000 (RSLCnano) chromatography system. Each sample was loaded onto Biobasic C18 PicofritTM column (100 mm length, 75 mm ID) and was separated by an increasing acetonitrile gradient, using a 30 min reverse phase gradient at a flow rate of 300 nL/min. The mass spectrometer was operated in positive ion mode with a capillary temperature of 200°C, a capillary voltage of 45 V, a tube lens voltage of 100 V and with a potential of 1900 V applied to the frit. All data were acquired with the mass spectrometer operating in automatic data dependent switching mode. A high resolution MS scan (300–2000 Dalton) was performed using the Orbitrap to select the seven most intense ions prior to MS/MS analysis using the ion trap. For peptide data analysis MS/MS data were searched using SEQUEST (BioWorks 3.3.1, Thermo Finnigan, UK) software against the Uniprot/Swiss-Prot human database (September 2010 release version 74; 513948 total database entries). The following criteria were used to search for protein identification: precursor mass ion tolerance (1.5 AMU), fragment ion tolerance (1 AMU), methionine oxidation, and carboxyamidomethylation modifications. Trypsin was specified as the proteolytic enzyme and up to two missing cleavages were allowed. The peptides were filtered according to the following parameters: Xcorr above 1.90 (+1), 2.50 (+2) and 3.20 (+3), peptide probability of *P*≤0.001.

**Gene ontology analysis**

Gene Ontology analysis was carried out using DAVID (<http://david.abcc.ncifcrf.gov>)^3, 4^ as previously described.^2^ FatGO terms from the functional annotation chart results with a significance level of *P*<0.001 and false discovery rate (FDR) <0.02 for cellular component, *P*<0.001 and FDR<0.4 for molecular function and *P*<0.001 and FDR<0.01 for biological processes were collated.^5^ The Kyoto Encyclopaedia of Genes and Genomes (KEGG) pathway database was also searched through DAVID to determine involvement in established biological pathways.

**Electroconvulsive stimulation (ECS)**

ECS was carried out as previously described.^6^ Briefly, male Sprague-Dawley rats (Harlan Laboratories, UK; weight on arrival, 150-200g) were housed four per cage and maintained in an ambient temperature of 20±1ºC with lighting on a 12 h light-dark cycle. Animals had free access to food and water and were fed a standard laboratory diet (Red Mills, Ireland). Experimental procedures were approved by the Bioresources Ethics Committee, Trinity College Dublin and were in compliance with the European Council Directive (86/609/EEC). Animals were handled for at least one week prior to experimentation to habituate them to the ear-clips used for electroconvulsive stimulation (ECS) and alleviate any stress caused by experimental handling. Animals were weight matched and randomized to receive either real or sham ECS. Animals received bilateral ECS thrice weekly (Monday, Wednesday, and Friday) via moistened pads on spring-loaded ear clip electrodes using a pulse generator (ECT unit 57800-001, Ugo Basile, Italy) and were weighed immediately prior to each treatment. Stimulation parameters were: frequency, 100 pulses/second; pulse width, 0.5ms; stimulus duration, 0.7s; current, 75mA. Sham animals had ear clip electrodes applied but did not receive an electrical stimulation. ECS treatment resulted in grand mal seizures with both tonic and clonic components. Animals were sacrificed under isoflurane anesthesia 4 h following either a single (acute) or the tenth (chronic) ECS treatment. Whole blood was harvested by cardiac puncture into K_2_EDTA coated syringes and 500μl was transferred to RNAprotect Animal Blood Tubes (Qiagen Ltd., Ireland). Large blood vessels were removed from the brain and the dentate gyrus, hippocampus, frontal cortex, and cerebellum were dissected out and snap-frozen on dry-ice. All samples were stored at -80°C prior to analysis. Laboratory analyses were performed with the investigator unblinded to the group the samples belonged to.

**mRNA extraction, cDNA synthesis and qRT-PCR**

Total RNA was extracted from rodent brain and blood samples using a mirVana miRNA Isolation kit (Ambion, Applied Biosystems, UK) and an RNeasy Protect Animal Blood Kit (Qiagen Ltd., Ireland). Following reverse transcription, using a high capacity cDNA archive kit (Applied Biosystems, UK), gene expression was assessed by quantitative real-time polymerase chain reaction (qRT-PCR) on a StepOnePlus^TM^ instrument (Applied Biosystems, UK) using TaqMan^®^ Gene Expression Assays and TaqMan^®^ Fast Advanced Master Mix (Applied Biosystems, UK). A master mix was made by combining 5μL of TaqMan Fast Advanced Master Mix with 0.5μL of each primer. 6μL of this master mix was added to each well in the PCR plate along with 4μL of cDNA. The cycling conditions consisted of an initial polymerase activation step of 95°C for 20 s followed by 40 cycles of 95°C for 1 s (denaturation) and 60°C for 20 s (transcription). Relative quantification (RQ) mRNA levels were calculated using the comparative CT method^7^ after normalization to glyceraldehyde 3-phosphate dehydrogenase (GAPDH). Assay IDs were as follows: Rat PEDF (SERPINF1), Rn00709999_m1; Rat GAPDH, Rn01775763.

**Supplementary Table 1.** Demographic and clinical characteristics of participants in the validation study

| **Characteristic** | **ECT-MDD**  **(*n*=57)** | **Controls**  **(*n*=43)** | **Statistical test** |
| --- | --- | --- | --- |
| Age, mean (SD), y | 52.6 (14.6) | 49.1 (14.1) | t=1.212, *P*=0.23 |
| Sex, No. (%) |  |  |  |
| Male | 21 (36.8) | 11 (25.6) | χ^2^=1.43, *P*=0.28 |
| Female | 36 (63.2) | 32 (74.4) |  |
| BMI, mean (SD) | 26.8 (5.1) | 24.8 (4.1) | t=2.15, *P*=0.034 |
| Alcohol. median units per week (range) | 0 (0-12) | 8 (0-20) | Mann-Whitney U=6.12, *P*<0.001 |
| Smokers, No. (%) | 27 (47.4) | 5 (11.6) | χ^2^=14.88, *P*<0.001 |
| Socio-economic Group, No. (%) |  |  |  |
| 1 | 12 (21.1) | 15 (34.9) |  |
| 2 | 6 (10.5) | 7 (16.3) |  |
| 3 | 15 (26.3) | 10 (23.3) | χ^2^=4.80, *P*=0.31 |
| 4 | 14 (24.6) | 5 (11.6) |  |
| 5 | 10 (17.5) | 6 (14.0) |  |
| Bipolar depression, No. (%) | 13 (22.8) |  |  |
| Psychotic depression, No. (%) | 11 (19.3) |  |  |
| Medications, n (%) taking |  |  |  |
| SSRI | 14 (24.6) |  |  |
| SNRI | 25 (43.9) |  |  |
| TCA | 16 (28.1) |  |  |
| MAOI | 1 (1.8) |  |  |
| Mirtazapine | 23 (40.4) |  |  |
| Trazodone | 4 (7.0) |  |  |
| Agomelatine | 1 (1.8) |  |  |
| Bupropion | 1 (1.8) |  |  |
| Buspirone | 1 (1.8) |  |  |
| Lithium | 18 (31.6) |  |  |
| Sodium Valproate | 3 (6.5) |  |  |
| Lamotrigine | 6 (10.5) |  |  |
| Antipsychotics | 43 (75.4) |  |  |
| Benzodiazepines | 32 (56.1) |  |  |
| Non-benzodiazepine hypnotics | 34 (59.6) |  |  |
| Pregabalin | 5 (8.8) |  |  |
| Baseline HAM-D, mean (SD) | 30.1 (7.1) | 3.1 (2.3) | t=26.89, *P*<0.001 |
| Post-ECT HAM-D, mean (SD) | 14.6 (8.5) |  |  |
| Electrode placement, No. (%) |  |  |  |
| Unilateral | 27 (47.4) |  |  |
| Bitemporal | 30 (52.6) |  |  |
| Number of ECT sessions, mean (SD) | 8.32 (2.4) |  |  |
| Remitters, No. (%) | 16 (28.1) |  |  |

Abbreviations: BMI, body mass index; ECT, electroconvulsive therapy; HAM-D, Hamilton depression rating scale, 24-item version;; mC, millicoulombs; EEG, electroencephalogram; SSRI, selective serotonin reuptake inhibitor; SNRI, serotonin-norepinephrine reuptake inhibitor; TCA, tricyclic antidepressant; MAOI, monoamine oxidase inhibitor


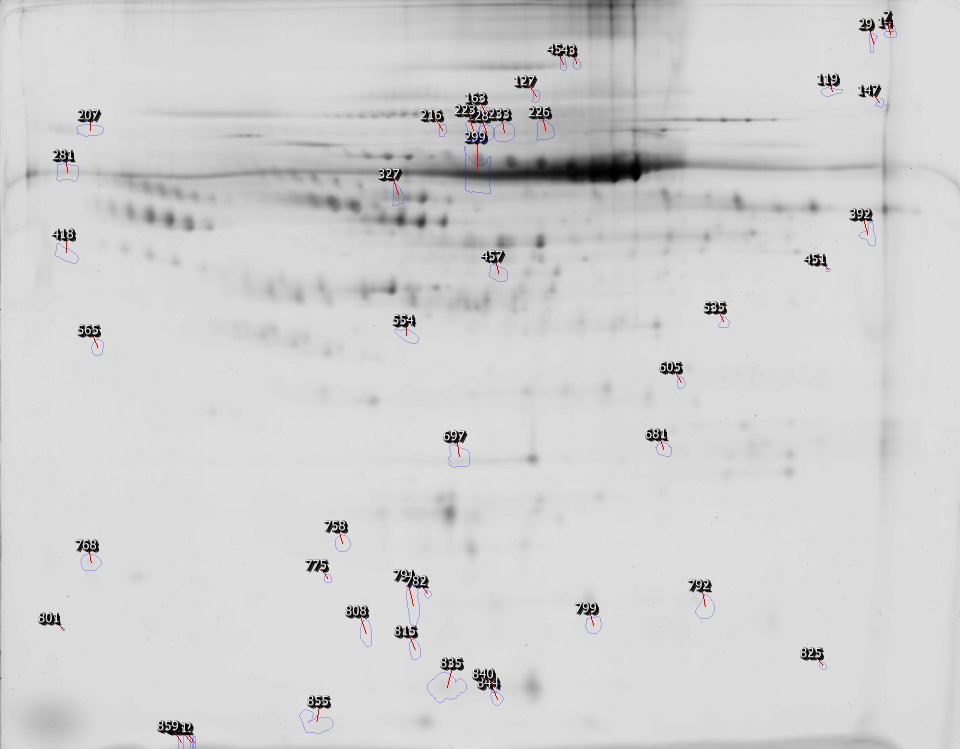


**Supplementary Figure 1.** Representative Low-abundance 2D-DIGE gel

**
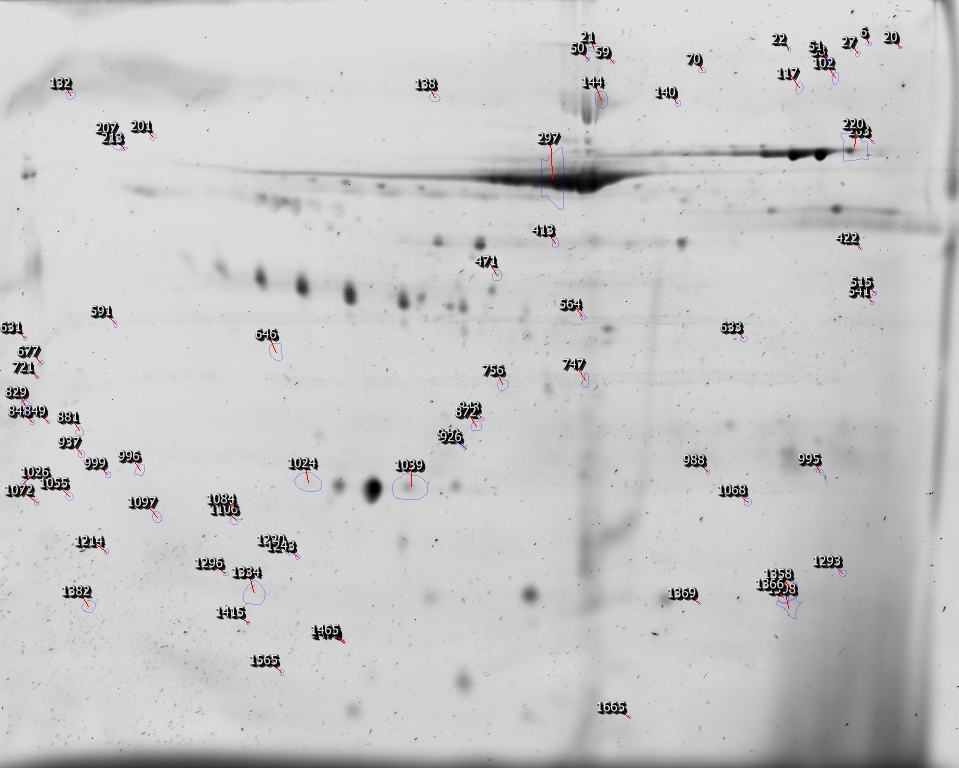
**

**Supplementary Figure 2.** Representative High-abundance 2D-DIGE gel

**Supplementary Table 2.** Low abundance proteins identified following 2D-DIGE and mass spectrometry

|  | | | | **Min 2 Unique Peptides** | | | | |
| --- | --- | --- | --- | --- | --- | --- | --- | --- |
| **Spot No.** | **Spot Fold-Change** | **Protein** | **Peptides** | ***P*-value (protein)** | **Protein Score** | **% sequence coverage** | **Molecular Weight** | **Accession No.** |
| **226** | **1.1** | **Tropomyosin alpha-1 chain** | K.LEEAEKAADESER.G  K.KATDAEADVASLNR.R  R.SKQLEDELVSLQKK.L  -.KLVIIESDLER.-  R.SKQLEDELVSLQK.K  -.SIDDLEDELYAQK.-  -.LATALQKLEEAEKAADESER.-  K.SIDDLEDELYAQK.L | **8.65E-09** | **80.20** | **25.35** | **32688.7** | **P09493** |
|  |  | **Complement C1r subcomponent** | K.QDAC#QGDSGGVFAVR.D  R.FC#GQLGSPLGNPPGK.K  R.ESEQGVYTC#TAQGIWK.N  R.LPVANPQAC#ENWLR.G  K.PYPNNFETTTVITVPTGYR.V  R.WVATGIVSWGIGC#SR.G | **3.49E-12** | **60.19** | **13.33** | **80066.8** | **P00736** |
|  |  | **Myosin light chain 3** | R.ALGQNPTQAEVLR.V  -.ITYGQC#GDVLR.-  K.NKDTGTYEDFVEGLR.V  K.LMAGQEDSNGC#INYEAFVK.H  R.LTEDEVEKLMAGQEDSNGC#INYEAFVK.H | **5.22E-14** | **50.27** | **33.85** | **21917.93** | **P08590** |
|  |  | **ATP synthase subunit alpha, mitochondrial** | R.ILGADTSVDLEETGR.V  R.NVQAEEMVEFSSGLK.G | **2.98E-10** | **20.21** | **5.42** | **59713.7** | **P25705** |
|  |  |  |  |  |  |  |  |  |
|  |  |  |  |  |  |  |  |  |
|  |  |  |  |  |  |  |  |  |
|  |  |  |  |  |  |  |  |  |
| **Spot No.** | **Spot Fold-Change** | **Protein** | **Peptides** | ***P*-value (protein)** | **Protein Score** | **% sequence coverage** | **Molecular Weight** | **Accession No.** |
| **228** | **1.1** | **Tropomyosin alpha-1 chain** | K.KATDAEADVASLNR.R  K.ATDAEADVASLNR.R  -.AQKDEEKM*EIQEIQLK.-  R.SKQLEDELVSLQKK.L  -.AQKDEEKMEIQEIQLK.-  R.RIQLVEEELDR.A  R.KLVIIESDLER.A  -.LATALQKLEEAEK.-  -.LVIIESDLER.-  -.LATALQKLEEAEKAADESER.-  K.SIDDLEDELYAQK.L  K.AISEELDHALNDM*TSI.-  R.AELSEGKC#AELEEELKTVTNNLK.S | **2.20E-13** | **130.24** | **48.59** | **32688.7** | **P09493** |
|  |  | **Myosin light chain 3** | R.ALGQNPTQAEVLR.V  -.LM*AGQEDSNGC#INYEAFVK.-  K.LMAGQEDSNGC#INYEAFVK.H  R.LTEDEVEKLM*AGQEDSNGC#INYEAFVK.H  K.DTGTYEDFVEGLR.V  R.LTEDEVEKLMAGQEDSNGC#INYEAFVK.H | **3.33E-15** | **60.26** | **27.18** | **21917.9** | **P08590** |
|  |  | **Creatine kinase M-type** | -.GQSIDDMIPAQK.-  K.TDLNHENLKGGDDLDPNYVLSSR.V  -.GGDDLDPNYVLSSR.-  R.LGSSEVEQVQLVVDGVK.L  -.LSVEALNSLTGEFKGK.-  K.LSVEALNSLTGEFK.G | **1.73E-09** | **60.18** | **17.85** | **43073.9** | **P06732** |
|  |  | **LIM domain-binding protein 3** | -.SASYNLSLTLQK.-  -.ILAQMTGTEFM*QDPDEEALRR.-  -.EMAQMYQMSLR.-  -.TSLADVC#FVEEQNNVYC#ER.-  K.AAQSQLSQGDLVVAIDGVNTDTMTHLEAQNK.I | **1.81E-09** | **50.19** | **12.93** | **77086.1** | **O75112** |
|  |  |  |  |  |  |  |  |  |
|  |  |  |  |  |  |  |  |  |
|  |  |  |  |  |  |  |  |  |
| **Spot No.** | **Spot Fold-Change** | **Protein** | **Peptides** | ***P*-value (protein)** | **Protein Score** | **% sequence coverage** | **Molecular Weight** | **Accession No.** |
| **228** | **1.1** | **ATP synthase subunit alpha, mitochondrial** | R.ILGADTSVDLEETGR.V  -.TGTAEMSSILEER.-  R.NVQAEEMVEFSSGLK.G  R.TGAIVDVPVGEELLGR.V | **6.97E-11** | **40.18** | **10.67** | **59713.7** | **P25705** |
|  |  | **ATP synthase subunit beta, mitochondrial** | K.AHGGYSVFAGVGER.T  -.VALVYGQMNEPPGAR.-  R.LVLEVAQHLGESTVR.T  -.FTQAGSEVSALLGR.- | **6.51E-11** | **40.18** | **10.96** | **56524.7** | **P06576** |
|  |  | **Complement C1r subcomponent** | K.QDAC#QGDSGGVFAVR.D  R.FC#GQLGSPLGNPPGK.K  -.ESEQGVYTC#TAQGIWK.-  R.LPVANPQAC#ENWLR.G | **3.53E-12** | **40.16** | **8.51** | **80066.8** | **P00736** |
|  |  | **Creatine kinase S-type, mitochondrial** | -.LSEMTEQDQQR.-  R.LGYILTC#PSNLGTGLR.A  R.GTGGVDTAAVADVYDISNIDR.I | **1.71E-11** | **30.23** | **11.46** | **47474.4** | **P17540** |
|  |  | **Stress-70 protein, mitochondrial** | K.SQVFSTAADGQTQVEIK.V  -.TTPSVVAFTADGER.-  K.GAVVGIDLGTTNSC#VAVMEGK.Q | **1.86E-11** | **30.22** | **7.66** | **73634.8** | **P38646** |
|  |  | **Phosphoglycerate kinase 1** | R.AHSSMVGVNLPQK.A  K.LGDVYVNDAFGTAHR.A  K.WNTEDKVSHVSTGGGASLELLEGK.V | **4.06E-09** | **30.18** | **12.47** | **44586.2** | **P00558** |
|  |  | **60 kDa heat shock protein, mitochondrial** | K.NAGVEGSLIVEK.I  K.LVQDVANNTNEEAGDGTTTATVLAR.S  R.AAVEEGIVLGGGC#ALLR.C | **1.63E-06** | **30.18** | **9.42** | **61016.5** | **P10809** |
|  |  | **Triosephosphate isomerase** | -.IAVAAQNC#YK.-  R.HVFGESDELIGQK.V  K.VTNGAFTGEISPGMIK.D | **1.20E-11** | **30.15** | **15.66** | **26652.7** | **P60174** |
|  |  | **Myosin-6** | -.KVQHELDEAEERADIAESQVNK.-  -.LQDLVDKLQLK.- | **1.55E-06** | **20.20** | **1.70** | **223593.9** | **P13533** |
|  |  | **Desmin OS=Homo sapiens** | R.FLEQQNAALAAEVNR.L  -.HQIQSYTC#EIDALKGTNDSLMR.- | **7.88E-05** | **20.19** | **7.87** | **53503.2** | **P17661** |
|  |  | **Peptidyl-prolyl cis-trans isomerase A** | -.VKEGMNIVEAMER.-  -.SIYGEKFEDENFILK.- | **1.02E-10** | **20.19** | **16.97** | **18000.9** | **P62937** |
| **Spot No.** | **Spot Fold-Change** | **Protein** | **Peptides** | ***P*-value (protein)** | **Protein Score** | **% sequence coverage** | **Molecular Weight** | **Accession No.** |
| **228** | **1.1** | **NADH dehydrogenase [ubiquinone] flavoprotein 2, mitochondrial** | -.YHIQVC#TTTPC#MLR.-  R.VYEVATFYTMYNR.K | **5.90E-09** | **20.19** | **10.84** | **27374.0** | **P19404** |
|  |  | **Vinculin** | -.LVQAAQMLQSDPYSVPARDYLIDGSR.-  -.GILEYLTVAEVVETMEDLVTYTK.- | **1.75E-13** | **20.18** | **4.32** | **123721.9** | **P18206** |
|  |  | **Haptoglobin** | -.TEGDGVYTLNNEK.-  K.YVM*LPVADQDQC#IR.H | **2.16E-07** | **20.17** | **6.65** | **45176.6** | **P00738** |
|  |  | **Troponin I, cardiac muscle** | K.NIDALSGMEGR.K  -.NITEIADLTQK.- | **4.21E-07** | **20.15** | **10.48** | **23992.8** | **P19429** |
|  |  | **Fumarate hydratase, mitochondrial** | R.IYELAAGGTAVGTGLNTR.I  -.SGLGELILPENEPGSSIMPGK.- | **4.72E-08** | **20.15** | **7.65** | **54602.2** | **P07954** |
|  |  | **Haptoglobin-related protein** | -.TEGDGVYTLNDKK.-  K.SC#AVAEYGVYVK.V | **3.60E-06** | **18.15** | **7.18** | **39004.7** | **P00739** |
| **233** | **1.1** | **Myosin light chain 3** | -.ALGQNPTQAEVLR.-  R.ALGQNPTQAEVLR.V  -.NKDTGTYEDFVEGLR.-  K.LMAGQEDSNGC#INYEAFVK.H  -.LTEDEVEKLM*AGQEDSNGC#INYEAFVK.-  -.DTGTYEDFVEGLR.-  -.IKIEFTPEQIEEFK.-  R.LTEDEVEKLMAGQEDSNGC#INYEAFVK.H | **1.00E-30** | **80.29** | **35.38** | **21917.9** | **P08590** |
|  |  | **Tropomyosin alpha-1 chain** | K.KATDAEADVASLNR.R  K.ATDAEADVASLNR.R  R.SKQLEDELVSLQK.K  -.IQLVEEELDR.-  -.SIDDLEDELYAQK.-  -.LATALQKLEEAEKAADESER.-  K.SIDDLEDELYAQK.L  -.AELSEGKC#AELEEELKTVTNNLK.- | **2.47E-10** | **80.22** | **32.75** | **32688.7** | **P09493** |
| **Spot No.** | **Spot Fold-Change** | **Protein** | **Peptides** | ***P*-value (protein)** | **Protein Score** | **% sequence coverage** | **Molecular Weight** | **Accession No.** |
| **233** | **1.1** | **Complement C1r subcomponent** | -.QDAC#QGDSGGVFAVR.-  R.FC#GQLGSPLGNPPGK.K  R.ESEQGVYTC#TAQGIWK.N  R.LPVANPQAC#ENWLR.G  -.WILTAAHTLYPK.-  -.LFGEVTSPLFPK.- | **6.36E-10** | **60.18** | **11.91** | **80066.8** | **P00736** |
|  |  | **Creatine kinase M-type** | -.LGSSEVEQVQLVVDGVK.-  -.SFLVWVNEEDHLR.-  R.GIWHNDNKSFLVWVNEEDHLR.V  -.LSVEALNSLTGEFK.- | **1.89E-09** | **40.21** | **13.65** | **43073.9** | **P06732** |
|  |  | **Creatine kinase S-type, mitochondrial** | -.GTGGVDTAAVADVYDISNIDR.-  K.TFLIWINEEDHTR.V  R.GWEFMWNER.L  R.SEVELVQIVIDGVNYLVDC#EK.K | **9.11E-11** | **40.21** | **15.27** | **47474.4** | **P17540** |
|  |  | **Myosin regulatory light chain 2, ventricular/car-diac muscle isoform** | K.LKGADPEETILNAFK.V  -.VNVKNEEIDEMIKEAPGPINFTVFLTMFGEK.-  -.EAPGPINFTVFLTMFGEK.- | **2.22E-15** | **30.26** | **27.71** | **18777.4** | **P10916** |
|  |  | **ATP synthase subunit beta, mitochondrial** | -.VALVYGQMNEPPGAR.-  -.LVLEVAQHLGESTVR.-  R.VALTGLTVAEYFRDQEGQDVLLFIDNIFR.F | **1.05E-09** | **30.21** | **11.15** | **56524.7** | **P06576** |
|  |  | **Stress-70 protein, mitochondrial** | K.SQVFSTAADGQTQVEIK.V  K.GAVVGIDLGTTNSC#VAVMEGK.Q | **6.56E-08** | **20.22** | **5.60** | **73634.8** | **P38646** |
|  |  | **LIM domain-binding protein 3** | -.SASYNLSLTLQK.-  K.AAQSQLSQGDLVVAIDGVNTDTMTHLEAQNK.I | **7.49E-08** | **20.21** | **5.91** | **77086.1** | **O75112** |
|  |  | **ATP synthase subunit alpha, mitochondrial** | R.ILGADTSVDLEETGR.V  R.NVQAEEMVEFSSGLK.G | **9.90E-11** | **20.20** | **5.42** | **59713.7** | **P25705** |
|  |  | **60 kDa heat shock protein, mitochondrial** | -.LVQDVANNTNEEAGDGTTTATVLAR.-  R.AAVEEGIVLGGGC#ALLR.C | **5.44E-07** | **20.17** | **7.33** | **61016.5** | **P10809** |
| **Spot No.** | **Spot Fold-Change** | **Protein** | **Peptides** | ***P*-value (protein)** | **Protein Score** | **% sequence coverage** | **Molecular Weight** | **Accession No.** |
| **281** | **-1.2** | **Alpha-1B-glycoprotein** | -.HQFLLTGDTQGR.-  -.PPFGGSAPSER.-  K.HQFLLTGDTQGR.Y  -.ATWSGAVLAGR.-  R.IFFHLNAVALGDGGHYTC#R.Y  R.IFFHLNAVALGDGGHYTCR.Y  R.TPGAAANLELIFVGPQHAGNYR.C  K.SLPAPWLSMAPVSWITPGLK.T | **1.22E-14** | **80.23** | **19.19** | **54238.7** | **P04217** |
|  |  | **Complement factor B** | K.VSEADSSNADWVTK.Q  R.FLC#TGGVSPYADPNTC#R.G  -.YGLVTYATYPK.- | **1.63E-09** | **30.18** | **5.50** | **85478.6** | **P00751** |
|  |  | **Serum albumin** | K.TC#VADESAENC#DK.S  -.KVPQVSTPTLVEVSR.-  K.VPQVSTPTLVEVSR.N | **4.14E-08** | **30.16** | **4.60** | **69321.6** | **P02768** |
|  |  | **ATP synthase subunit alpha, mitochondrial** | -.ILGADTSVDLEETGR.-  R.NVQAEEMVEFSSGLK.G | **1.30E-09** | **20.21** | **5.42** | **59713.7** | **P25705** |
| **299** | **-1.1** | **Serum albumin** | K.TC#VADESAENC#DK.S  K.VHTEC#C#HGDLLEC#ADDR.A  -.KVPQVSTPTLVEVSR.-  -.VPQVSTPTLVEVSR.-  -.PLVEEPQNLIK.-  R.RHPDYSVVLLLR.L  -.QNC#ELFEQLGEYK.-  K.VFDEFKPLVEEPQNLIK.Q  -.AVMDDFAAFVEK.- | **1.10E-10** | **90.19** | **16.26** | **69321.6** | **P02768** |
|  |  | **Alpha-1B-glycoprotein** | K.HQFLLTGDTQGR.Y  -.NGVAQEPVHLDSPAIK.-  -.ATWSGAVLAGR.-  -.SGLSTGWTQLSK.-  -.IFFHLNAVALGDGGHYTC#R.-  R.TPGAAANLELIFVGPQHAGNYR.C | **1.11E-15** | **60.23** | **18.59** | **54238.7** | **P04217** |
|  |  | **Myosin light chain 3** | -.NKDTGTYEDFVEGLR.-  K.LMAGQEDSNGC#INYEAFVK.H  R.LTEDEVEKLMAGQEDSNGC#INYEAFVK.H | **2.55E-14** | **30.22** | **21.54** | **21917.9** | **P08590** |
| **Spot No.** | **Spot Fold-Change** | **Protein** | **Peptides** | ***P*-value (protein)** | **Protein Score** | **% sequence coverage** | **Molecular Weight** | **Accession No.** |
| **299** | **-1.1** | **Fibrinogen alpha chain** | R.GSESGIFTNTK.E  -.HPDEAAFFDTASTGK.-  K.GLIDEVNQDFTNR.I | **1.40E-08** | **30.14** | **4.50** | **94914.3** | **P02671** |
| **457** | **-1.1** | **Pigment epithelium-derived factor** | R.YGLDSDLSC#K.I  R.KTSLEDFYLDEER.T  K.TSLEDFYLDEER.T  K.LQSLFDSPDFSK.I  K.LAAAVSNFGYDLYR.V | **3.43E-09** | **50.17** | **11.72** | **46283.4** | **P36955** |
|  |  | **Fibrinogen gamma chain** | K.VAQLEAQC#QEPC#K.D  -.DTVQIHDITGK.-  R.YLQEIYNSNNQK.I | **4.64E-08** | **30.17** | **7.95** | **51478.9** | **P02679** |
|  |  | **Complement factor I** | R.GLETSLAEC#TFTK.R  R.EANVAC#LDLGFQQGADTQR.R | **2.30E-07** | **20.19** | **5.49** | **65676.7** | **P05156** |
| **681** | **1.2** | **Serum amyloid P-component** | K.IVLGQEQDSYGGK.F  R.VGEYSLYIGR.H  R.AYSLFSYNTQGR.D  R.AYSLFSYNTQGR.D | **2.46E-09** | **30.16** | **15.70** | **25371.1** | **P02743** |
| **697** | **1.3** | **Serum amyloid P-component** | K.IVLGQEQDSYGGK.F  -.VGEYSLYIGR.- | **3.59E-08** | **30.14** | **15.70** | **25371.1** | **P02743** |
| **835** | **1.2** | **Haptoglobin** | -.TEGDGVYTLNNEK.-  -.LPEC#EADDGC#PKPPEIAHGYVEHSVR.-  K.PPEIAHGYVEHSVR.Y | **3.00E-10** | **30.17** | **13.05** | **45176.6** | **P00738** |
| **855** | **1.0** | **Transthyretin** | -.KAADDTWEPFASGK.-  K.AADDTWEPFASGK.T  R.GSPAINVAVHVFR.K | **2.86E-09** | **30.18** | **18.37** | **15877.1** | **P02766** |

**Supplementary Table 3.** High abundance proteins identified following 2D-DIGE and mass spectrometry

|  | | | | **Min 2 Unique Peptides** | | | | |
| --- | --- | --- | --- | --- | --- | --- | --- | --- |
| **Spot No.** | **Spot Fold-Change** | **Protein** | **Peptides** | ***P*-value (protein)** | **Protein Score** | **% sequence coverage** | **Molecular Weight** | **Accession No.** |
| **220** | **1.2** | **Serotransferrin** | R.KPVEEYANC#HLAR.A  K.PVEEYANC#HLAR.A  K.DSGFQM*NQLR.G  R.WC#AVSEHEATK.C  K.DC#HLAQVPSHTVVAR.S  K.DGAGDVAFVK.H  K.LC#M*GSGLNLC#EPNNK.E  K.IEC#VSAETTEDCIAK.I  K.C#DEWSVNSVGK.I  K.DSGFQMNQLR.G  K.EGYYGYTGAFR.C  K.KSASDLTWDNLK.G  K.LC#MGSGLNLC#EPNNK.E  K.SASDLTWDNLK.G  K.EFQLFSSPHGK.D  K.HSTIFENLANK.A  R.FDEFFSEGC#APGSK.K  R.DQYELLC#LDNTR.K  K.DYELLC#LDGTR.K  K.IM*NGEADAM*SLDGGFVYIAGK.C  K.EDPQTFYYAVAVVK.K  K.MYLGYEYVTAIR.N  R.TAGWNIPM*GLLYNK.I  K.IM*NGEADAMSLDGGFVYIAGK.C  -.IMNGEADAM*SLDGGFVYIAGK.-  K.IMNGEADAMSLDGGFVYIAGK.C  R.TAGWNIPMGLLYNK.I  R.SAGWNIPIGLLYC#DLPEPR.K | **3.86E-13** | **278.26** | **37.54** | **77013.7** | **P02787** |
|  |  |  |  |  |  |  |  |  |
|  |  |  |  |  |  |  |  |  |
|  |  |  |  |  |  |  |  |  |
| **Spot No.** | **Spot Fold-Change** | **Protein** | **Peptides** | ***P*-value (protein)** | **Protein Score** | **% sequence coverage** | **Molecular Weight** | **Accession No.** |
| **220** | **1.2** | **Serum albumin** | K.TC#VADESAENC#DK.S  K.YIC#ENQDSISSK.L  K.C#C#TESLVNR.R  K.VHTEC#C#HGDLLEC#ADDR.A  R.FKDLGEENFK.A  K.LVNEVTEFAK.T  K.VPQVSTPTLVEVSR.N  R.RPC#FSALEVDETYVPK.E  R.LVRPEVDVM*C#TAFHDNEETFLK.K  K.AVM*DDFAAFVEK.C  K.QNC#ELFEQLGEYK.F  -.LVRPEVDVMC#TAFHDNEETFLKK.-  R.LVRPEVDVMC#TAFHDNEETFLK.K  R.HPDYSVVLLLR.L  R.RHPDYSVVLLLR.L  K.AVMDDFAAFVEK.C  K.VFDEFKPLVEEPQNLIK.Q  R.LVRPEVDVMCTAFHDNEETFLK.K  K.SHC#IAEVENDEM*PADLPSLAADFVESK.D  K.SHC#IAEVENDEMPADLPSLAADFVESK.D  R.RHPYFYAPELLFFAK.R  K.DVFLGM*FLYEYAR.R  K.DVFLGMFLYEYAR.R | **1.17E-12** | **230.32** | **38.26** | **69321.6** | **P02768** |
|  |  | **Ig mu chain C region** | K.QVGSGVTTDQVQAEAK.E  K.YVTSAPM*PEPQAPGR.Y  K.YVTSAPMPEPQAPGR.Y  K.LIC#QATGFSPR.Q  K.YAATSQVLLPSK.D  K.GVALHRPDVYLLPPAR.E  R.VFAIPPSFASIFLTK.S | **5.76E-08** | **70.23** | **18.81** | **49275.6** | **P01871** |
|  |  | **Apolipoprotein A-I** | K.DLATVYVDVLK.D  R.EQLGPVTQEFWDNLEK.E | **2.30E-08** | **20.15** | **10.11** | **30758.9** | **P02647** |
| **Spot No.** | **Spot Fold-Change** | **Protein** | **Peptides** | ***P*-value (protein)** | **Protein Score** | **% sequence coverage** | **Molecular Weight** | **Accession No.** |
| **297** | **1.1** | **Serum albumin** | K.TC#VADESAENCDK.S  K.ADDKETC#FAEEGKK.L  K.TC#VADESAENC#DK.S  K.ADDKETCFAEEGKK.L  K.C#C#TESLVNR.R  K.AAFTEC#C#QAADK.A  K.EC#C#EKPLLEK.S  K.AAFTECC#QAADK.A  -.AAFTEC#CQAADK.-  K.YICENQDSISSK.L  K.YIC#ENQDSISSK.L  K.VHTEC#C#HGDLLEC#ADDR.A  R.FKDLGEENFK.A  K.VHTEC#CHGDLLEC#ADDR.A  K.KYLYEIAR.R  K.YLYEIAR.R  K.LVNEVTEFAK.T  K.KQTALVELVK.H  K.VPQVSTPTLVEVSR.N  K.KVPQVSTPTLVEVSR.N  R.LVRPEVDVM*C#TAFHDNEETFLKK.Y  K.SLHTLFGDK.L  K.KLVAASQAALGL.-  R.RPC#FSALEVDETYVPK.E  R.LVRPEVDVM*C#TAFHDNEETFLK.K  K.AVM*DDFAAFVEK.C  R.RHPDYSVVLLLR.L  R.LVRPEVDVMC#TAFHDNEETFLKK.Y  K.QNC#ELFEQLGEYK.F  R.LVRPEVDVMC#TAFHDNEETFLK.K  K.QNCELFEQLGEYK.F  K.AVMDDFAAFVEK.C  K.VFDEFKPLVEEPQNLIK.Q  R.LVRPEVDVMCTAFHDNEETFLK.K  K.SHC#IAEVENDEM*PADLPSLAADFVESK.D  -.SHC#IAEVENDEM*PADLPSLAADFVESK.-  K.SHC#IAEVENDEMPADLPSLAADFVESK.D  K.DVFLGM*FLYEYAR.R  K.DVFLGMFLYEYAR.R | **1.44E-14** | **388.32** | **47.95** | **69321.6** | **P02768** |
| **Spot No.** | **Spot Fold-Change** | **Protein** | **Peptides** | ***P*-value (protein)** | **Protein Score** | **% sequence coverage** | **Molecular Weight** | **Accession No.** |
| **1024** | **1.2** | **Serum albumin** | K.TC#VADESAENC#DK.S  K.YIC#ENQDSISSK.L  K.LVNEVTEFAK.T  K.QNC#ELFEQLGEYK.F  R.RHPDYSVVLLLR.L  K.AVMDDFAAFVEK.C  K.VFDEFKPLVEEPQNLIK.Q  K.SHC#IAEVENDEM*PADLPSLAADFVESK.D  K.DVFLGM*FLYEYAR.R  K.ALVLIAFAQYLQQC#PFEDHVK.L  K.DVFLGMFLYEYAR.R | **6.55E-10** | **110.26** | **24.63** | **69321.6** | **P02768** |
|  |  | **Apolipoprotein A-I** | R.DYVSQFEGSALGK.Q  K.LLDNWDSVTSTFSK.L  K.DLATVYVDVLK.D  R.EQLGPVTQEFWDNLEK.E  K.VSFLSALEEYTK.K | **7.01E-10** | **50.19** | **24.72** | **30758.9** | **P02647** |
|  |  | **Serotransferrin** | K.LC#MGSGLNLC#EPNNK.E  K.DYELLC#LDGTR.K  K.EDPQTFYYAVAVVK.K | **1.00E-05** | **30.16** | **5.73** | **77013.7** | **P02787** |
| **1039** | **1.2** | **Apolipoprotein A-I** | K.ATEHLSTLSEK.A  K.WQEEM*ELYR.Q  K.WQEEMELYR.Q  R.DYVSQFEGSALGK.Q  K.LLDNWDSVTSTFSK.L  K.VSFLSALEEYTKK.L  K.LREQLGPVTQEFWDNLEK.E  K.VSFLSALEEYTK.K  R.EQLGPVTQEFWDNLEK.E  R.QGLLPVLESFK.V | **6.07E-11** | **100.26** | **33.33** | **30758.9** | **P02647** |

**
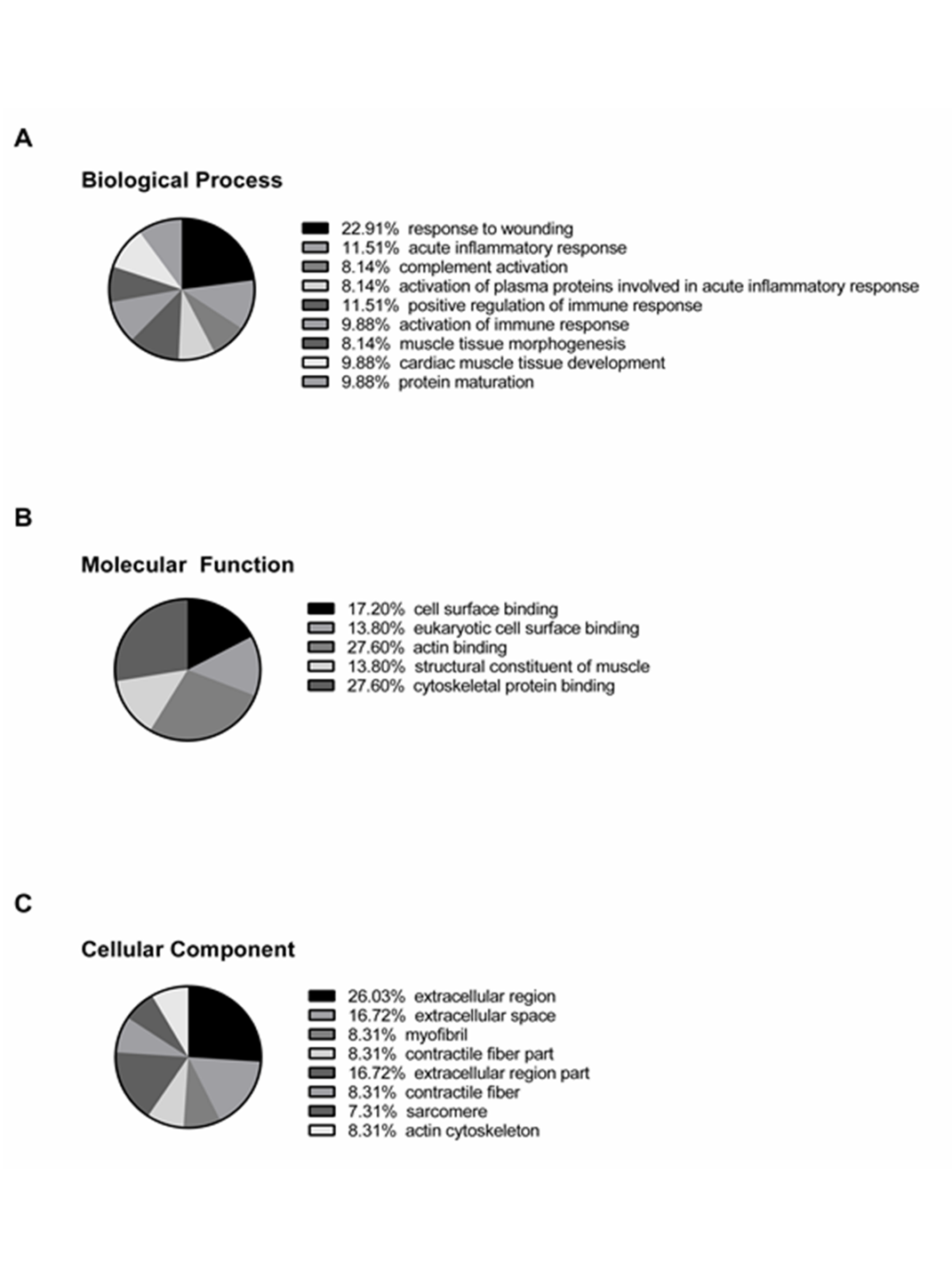
**

**Supplementary Figure 3.** DAVID classification of low-abundance proteins according to gene ontology (GO) terms

(A) biological process, (B) molecular function, and (C) cellular component. Percentages represent the proportion of proteins identified under each enrichment term.

**Supplementary Table 4.** Gene ontology (GO) analysis of low-abundance proteins

|  | ***P*-value** | **FDR** | **Accession numbers** |
| --- | --- | --- | --- |
| **Cellular component** | | | |
| extracellular region | 1.04E-10 | 1.22E-7 | P18206, P04217, P10643, P36980, P00751, P36955, P01008, P00739, P00738, P05160, P00736, P02671, P02679, P02787, P01042, P02768, Q03591, P02766, P21333, P02743, P62937, P06396, P10809, P05156, P02790 |
| extracellular space | 5.6E-10 | 6.62E-7 | P02679, P02671, P02787, P01042, P02768, Q03591, P02766, P02743, P36955, P06396, P10809, P05156, P01008, P00739, P00738, P02790 |
| Myofibril | 3.76E-8 | 4.45E-5 | P19429, P09493, P18206, P10916, O75112, P08590, P13533, P17661 |
| contractile fiber part | 4.26E-8 | 5.03E-5 | P19429, P09493, P18206, P10916, O75112, P08590, P13533, P17661 |
| extracellular region part | 5.545E-8 | 6.55E-5 | P02679, P02671, P02787, P01042, P02768, Q03591, P02766, P02743, P36955, P06396, P10809, P05156, P01008, P00739, P00738, P02790 |
| contractile fiber | 6.85E-8 | 8.09E-5 | P19429, P09493, P18206, P10916, O75112, P08590, P13533, P17661 |
| Sarcomere | 4.63E-7 | 5.47E-4 | P19429, P09493, P10916, O75112, P08590, P13533, P17661 |
| actin cytoskeleton | 1.46E-5 | 0.017 | P19429, P09493, P18206, P10916, P06396, P08590, P13533, P21333 |
|  | | | |
|  | ***P*-value** | **FDR** | **Accession numbers** |
| **Molecular function** | | | |
| cell surface binding | 8.89E-7 | 0.001 | P02679, P02671, P06576, P10809, P25705 |
| eukaryotic cell surface binding | 1.09E-5 | 0.01 | P02679, P02671, P06576, P25705 |
| actin binding | 1.77E-5 | 0.021 | P19429, P09493, P18206, P10916, P06396, P08590, P13533, P21333 |
| structural constituent of muscle | 1.76E-4 | 0.21 | P09493, P10916, P08590, P13533 |
| cytoskeletal protein binding | 2.75E-4 | 0.33 | P19429, P09493, P18206, P10916, P06396, P08590, P13533, P21333 |
| **Biological process** | | | |
| response to wounding | 2.91e-11 | 4.39E-8 | P02679, P02671, P02787, P01042, Q03591, P02743, P10643, P09493, P00751, P06396, P05156, P01008, P05160, P00736 |
| acute inflammatory response | 5.92E-9 | 8.95E-6 | P02787, P00751, P05156, Q03591, P02743, P10643, P00736 |
| complement activation | 7.92E-8 | 1.2E-4 | P00751, P05156, Q03591, P10643, P00736 |
| activation of plasma proteins involved in acute inflammatory response | 8.94E-8 | 1.35E-4 | P00751, P05156, Q03591, P10643, P00736 |
|  |  |  |  |
|  |  |  |  |
|  |  |  |  |
|  |  |  |  |
|  |  |  |  |
|  | ***P*-value** | **FDR** | **Accession numbers** |
| **Biological process** | | | |
| positive regulation of immune response | 9.05E-8 | 1.37E-4 | P00751, P10809, P05156, Q03591, P02790, P10643, P00736 |
| activation of immune response | 1.58E-7 | 2.38E-4 | P00751, P10809, P05156, Q03591, P10643, P00736 |
| ventricular cardiac muscle morphogenesis | 2.99E-7 | 4.51E-4 | P19429, P09493, P10916, P08590, P13533 |
| cardiac muscle tissue development | 3.79E-7 | 5.72E-4 | P19404, P19429, P09493, P10916, P08590, P13533 |
| cardiac muscle tissue morphogenesis | 7.1E-7 | 0.001 | P19429, P09493, P10916, P08590, P13533 |
| muscle tissue morphogenesis | 7.1E-7 | 0.001 | P19429, P09493, P10916, P08590, P13533 |
| protein maturation | 7.42E-7 | 0.001 | P00751, P10809, P05156, Q03591, P10643, P00736 |
| **KEGG Pathway** | | | |
| Complement and coagulation cascades | 1.37E-9 | 1.14E-6 | P02679, P02671, P00751, P01042, P05156, P01008, P05160, P10643, P00736 |

Classification is according to the Gene Ontology terms biological process, molecular function, and cellular component.

Abbreviation: FDR, false discovery rate.

**Supplementary Table 5.** Gene ontology classification of high-abundance proteins

|  | ***P*-value** | **FDR** | **Accession numbers** |
| --- | --- | --- | --- |
| **Cellular Component** | | | |
| extracellular region | 6.32E-5 | 0.06 | P01834, P02787, P01871, P02768, P00739, P00738, P01024, P02647, P06312 |
| extracellular space | 8.3E-4 | 0.81 | P02787, P02768, P00739, P00738, P01024, P02647 |
| **Molecular Function** | | | |
|  | ***P*-value** | **FDR** | **Accession numbers** |
| antigen binding | 6.27E-6 | 0.006 | P01834, P01871, P01876, P06312 |
| **Biological Process** | | | |
|  | ***P*-value** | **FDR** | **Accession numbers** |
| immune response | 6.88E-4 | 0.9 | P01834, P01871, P01876, P01024, P06312 |

Classification is according to the Gene Ontology terms biological process, molecular function, and cellular component.

Abbreviation: FDR, false discovery rate.

**Supplementary Table 6.** Clinical and demographic characteristics for enriched PEDF protein group

| **Characteristic** | **Total Group**  **(*n*=87)** |
| --- | --- |
| Age, mean (SD), y | 55.5 (14.6) |
| Sex, No. (%) |  |
| Male | 33 (37.9) |
| Female | 54 (62.1) |
| BMI, mean (SD) | 26.5 (4.9) |
| Alcohol. median units per week (range) | 0 (0-20) |
| Smokers, No. (%) | 38 (44.7) |
| Socio-economic Group, No. (%) |  |
| 1 | 19 (21.8) |
| 2 | 14 (16.1) |
| 3 | 21 (24.1) |
| 4 | 15 (17.2) |
| 5 | 18 (20.7) |
| Bipolar depression, No. (%) | 18 (20.7) |
| Psychotic depression, No. (%) | 18 (20.7) |
| Medications, n (%) taking |  |
| SSRI | 20 (23.0) |
| SNRI | 45 (51.7) |
| TCA | 23 (26.4) |
| Mirtazapine | 11 (12.6) |
| MAOI | 1 (1.1) |
| Trazodone | 7 (8.0) |
| Agomelatine | 1 (1.1) |
| Bupropion | 1 (1.1) |
| Buspirone | 1 (1.1) |
| Lithium | 31 (35.6) |
| Sodium Valproate | 6 (6.9) |
| Lamotrigine | 6 (6.9) |
| Antipsychotics | 62 (71.3) |
| Benzodiazepines | 55 (63.2) |
| Non-benzodiazepine hypnotics | 50 (57.5) |
| Pregabalin | 5 (5.7) |
| Baseline HAM-D, mean (SD) | 30.7 (7.1) |
| Post-ECT HAM-D, mean (SD) | 11.2 (8.5) |
| Electrode placement, No. (%) |  |
| Unilateral | 42 (48.3) |
| Bitemporal | 45 (51.7) |
| Number of ECT sessions, mean (SD) | 7.9 (2.4) |
| Remitters, No. (%) | 46 (52.9) |

Abbreviations: BMI, body mass index; ECT, electroconvulsive therapy; HAM-D, Hamilton depression rating scale; EOT, end of treatment; mC, millicoulombs; EEG, electroencephalogram; SSRI, selective serotonin reuptake inhibitor; SNRI, serotonin-norepinephrine reuptake inhibitor; TCA, tricyclic antidepressant; MAOI, monoamine oxidase inhibitor.

**References**

1. Glaviano A, O'Donovan SM, Ryan K, O'Mara S, Dunn MJ, McLoughlin DM. Acute phase plasma proteins are altered by electroconvulsive stimulation. *J Psychopharmacol* 2014; **28**(12)**:** 1125-1134.

2. O'Donovan SM, O'Mara S, Dunn MJ, McLoughlin DM. The persisting effects of electroconvulsive stimulation on the hippocampal proteome. *Brain Res* 2014; **1593:** 106-116.

3. Huang da W, Sherman BT, Lempicki RA. Systematic and integrative analysis of large gene lists using DAVID bioinformatics resources. *Nat Protoc* 2009; **4**(1)**:** 44-57.

4. Huang da W, Sherman BT, Lempicki RA. Bioinformatics enrichment tools: paths toward the comprehensive functional analysis of large gene lists. *Nucleic Acids Res* 2009; **37**(1)**:** 1-13.

5. Chitteti BR, Liu Y, Srour EF. Genomic and proteomic analysis of the impact of mitotic quiescence on the engraftment of human CD34+ cells. *PLOS One* 2011; **6**(3)**:** e17498.

6. Ryan KM, O'Donovan SM, McLoughlin DM. Electroconvulsive stimulation alters levels of BDNF-associated microRNAs. *Neuroscience Lett* 2013; **549:** 125-129.

7. Livak KJ, Schmittgen TD. Analysis of relative gene expression data using real-time quantitative PCR and the 2(-Delta Delta C(T)) Method. *Methods* 2001; **25**(4)**:** 402-408.
